# Supplementary material for: Simultaneous dendritic voltage and calcium imaging and somatic recording from Purkinje neurons in awake mice
Source: Nat Commun. 2018 Aug 23;9:3388. doi: 10.1038/s41467-018-05900-3 (PMC6107665; doi:10.1038/s41467-018-05900-3)
Supplement: Supplementary file 1 — Supplementary Information [file 41467_2018_5900_MOESM1_ESM.pdf]

Supplementary Figures

Simultaneous dendritic voltage and calcium imaging and somatic recording from Purkinje neurons in awake mice

Authors: Christopher J. Roome[1*], Bernd Kuhn[1*]

Affiliation: [1]Optical Neuroimaging Unit, Okinawa Institute of Science and Technology Graduate University (OIST), 1919-1 Tancha, Onna-son, Okinawa, 904-0495, Japan.

Supplementary Fig. 1 Simultaneous dendritic voltage and calcium imaging and somatic recording from Purkinje neurons in awake and anaesthetized mice.

Supplementary Fig. 2 Relationships between dendritic voltage and calcium signals during DCS events and simple spikes (SS) pause in awake mice.

Supplementary Fig. 3 Spatial variability of dendritic complex spikes.

Supplementary Fig. 4 Effect of 1% isoflurane anaesthesia on PN activity.

Supplementary Fig. 5 Cerebellar Purkinje neurons labelled in vivo with the voltage-sensitive dye ANNINE6-plus.

Supplementary Fig. 6 Long-term imaging of dendritic voltage from PNs of awake mice.

Supplementary Fig. 7 Imaging dendritic voltage at different dendritic locations of the same PNs in an awake mouse.

Supplementary Fig. 8 Supra-threshold and sub-threshold dendritic signals are blocked by Lidocaine.

Supplementary Fig. 9 Subthreshold dendritic hotspot activity correlates with somatic SS rate.

Supplementary Fig. 10 Two-photon excitation volume optimization for voltage imaging in vivo.

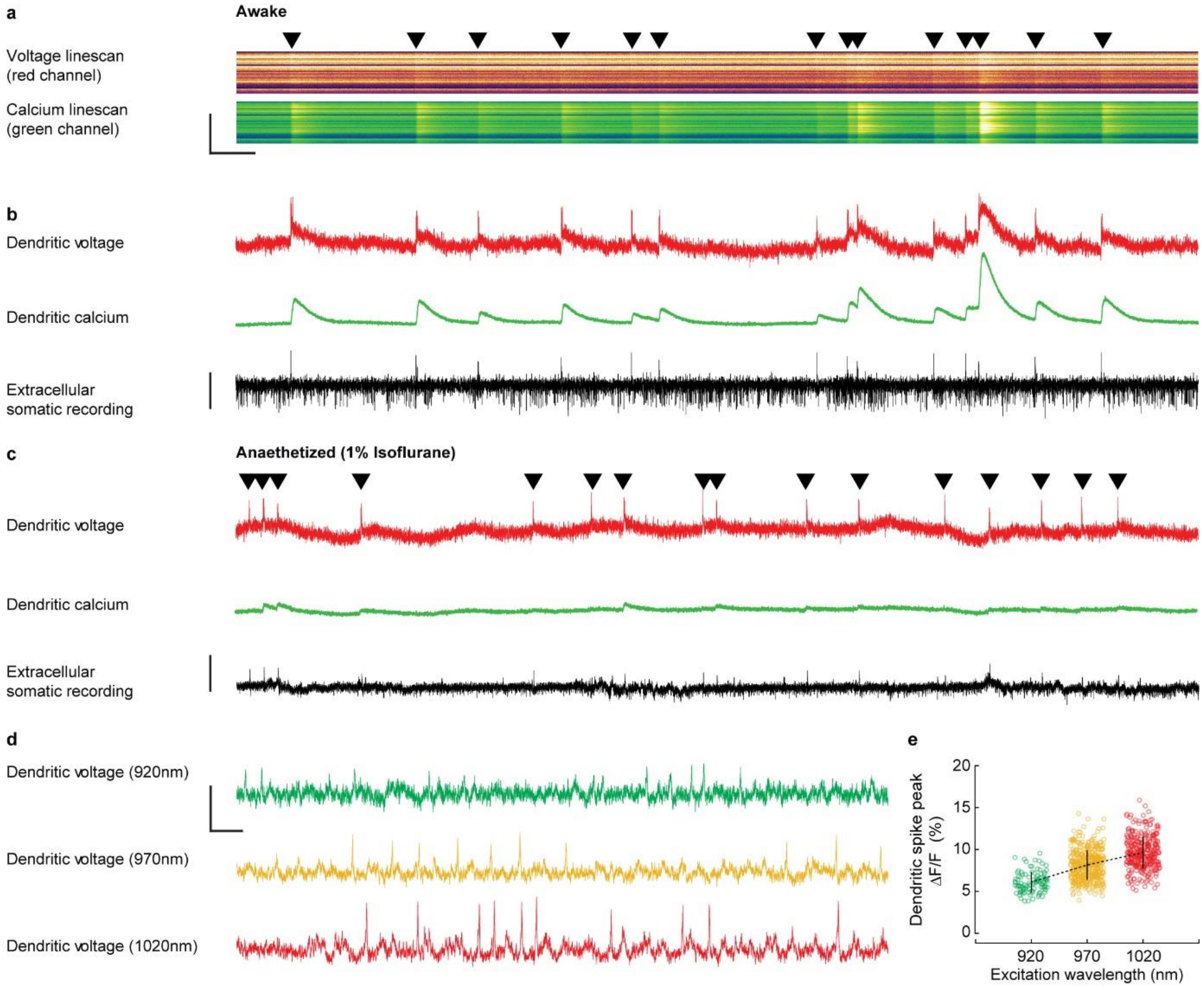

Supplementary Fig. 1 Simultaneous dendritic voltage and calcium imaging and somatic recording from Purkinje neurons in awake and anaesthetized mice. a Uncorrected 10 s linescans of dendritic voltage and calcium, with largest cross contamination of GCaMP6f fluorescence into the red channel. Closed triangles indicate dendritic complex spike (DCS) events. Vertical scale bar 150μm; horizontal scale bar 0.5s. b Corresponding traces of spatially averaged uncorrected dendritic voltage (red trace) and calcium (green trace) in arbitrary units, and simultaneous extracellular somatic recording (black trace, vertical scale bar 100pA). c Corresponding traces from the same PN during anesthesia (1% isoflurane). d Spatially averaged dendritic voltage with temporal filtering (5ms boxcar; to reduce noise for dendritic spike detection) recorded from the same Purkinje neuron at different excitation wavelengths (920nm green; 970nm yellow; 1020nm red). Vertical scale bar 10% ΔF/F; horizontal scale bar 50ms. e Dendritic spike peak amplitude measured during 500 seconds of recording for each of the excitation wavelengths shown in d. Note the wavelength dependence on peak amplitude (ΔF/F), confirming the optimal voltage sensitivity at the red spectral edge of absorption and the mechanism of voltage sensitivity. Also note that all dendritic spike amplitudes are slightly reduced in comparison to Fig. 2b due to temporal filtering. Bars show mean ± s.d.

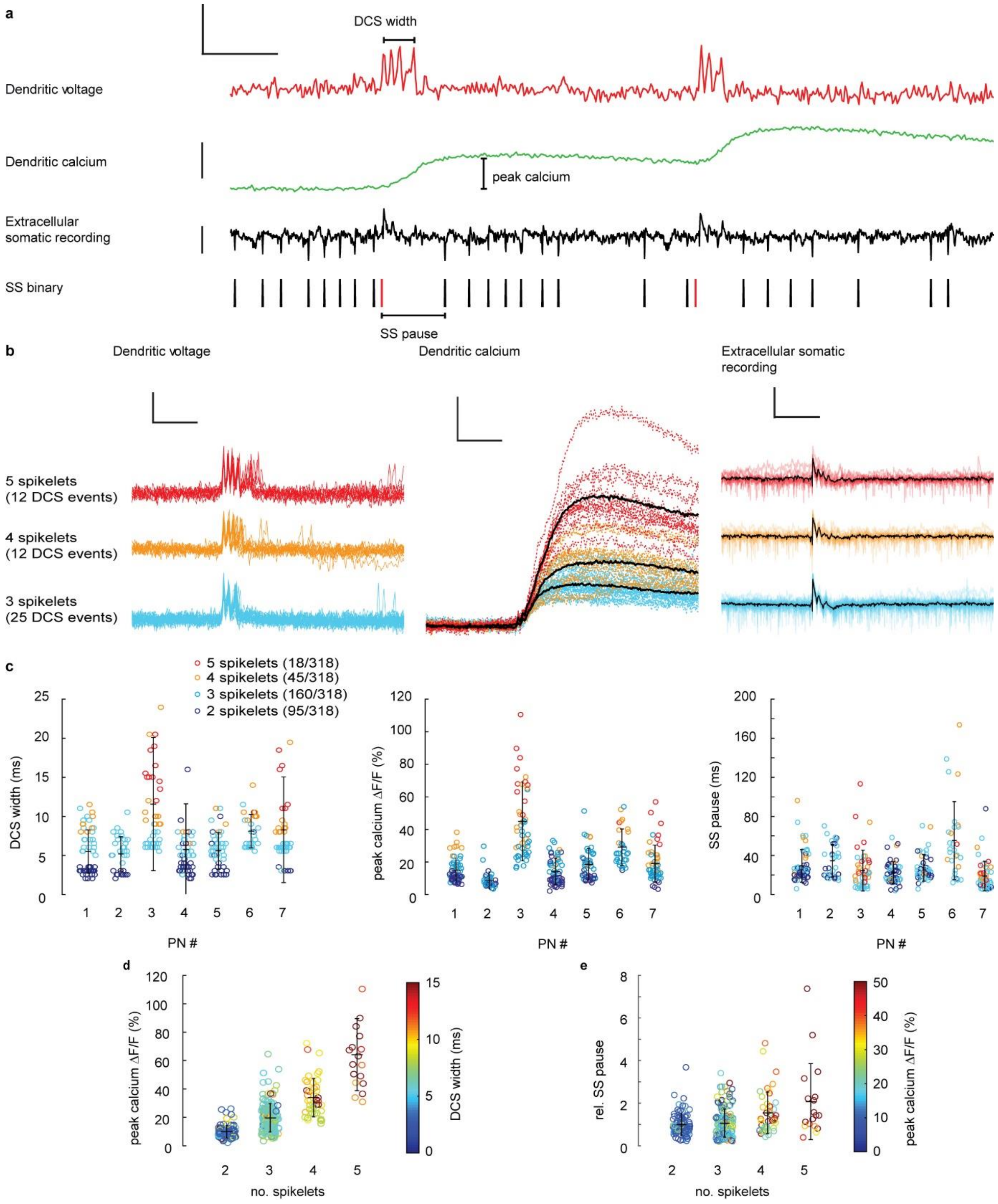

Supplementary Fig. 2 Relationships between dendritic voltage and calcium signals during DCS events and simple spikes (SS) pause in awake mice. a DCS width, peak calcium and SS pause measured as indicated. Voltage (red trace), vertical scale bar 20% ΔF/F; horizontal scale bar 25ms. Calcium (green trace), vertical scale bar 40% ΔF/F. Extracellular somatic recording (black trace), vertical scale bar 100pA. b All DCS events (voltage, vertical scale bar 10% ΔF/F), (calcium, vertical scale bar 20% ΔF/F), and corresponding somatic recording (vertical scale bar 100pA) from a single PN. Horizontal scale bars 25ms. Colors encode the number of dendritic spikelets. Black traces show average for each group. c DCS widths (left), peak calcium (middle), and corresponding SS pause (right), from 7 PNs (318 DCS events). d Relationship between number (no.) of spikelets in DCS, peak calcium and DCS width. e Relationship between no. of spikelets in DCS, relative SS pause (normalized to the average SS pause from the least no. of DCS spikelets for each PN) and the peak calcium. Bars show mean ± s.d.

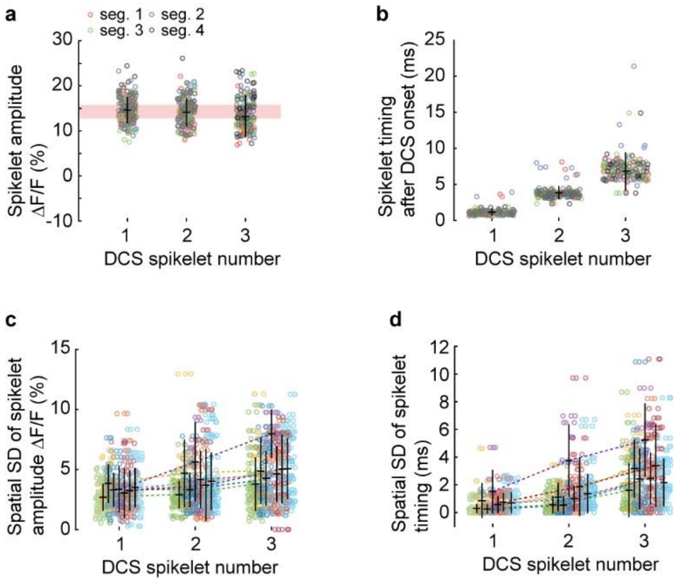

Supplementary Fig. 3 Spatial variability of dendritic complex spikes. a Amplitudes of the first three DCS spikelets measured in four dendritic segments (seg. 1-4) of the same PN. Pink bar shows expected error due to shot noise. b Spikelet timing after DCS onset for the first three spikelets measured in different segments (seg. 1-4) of the same PN dendrite. c Spatial standard deviation (SD) of spikelet amplitudes between dendritic segments and d spatial standard deviation (SD) of spikelet timing between dendritic segments, for all DCSs calculated from 7 PNs. Bars show mean ± s.d. for each PN.

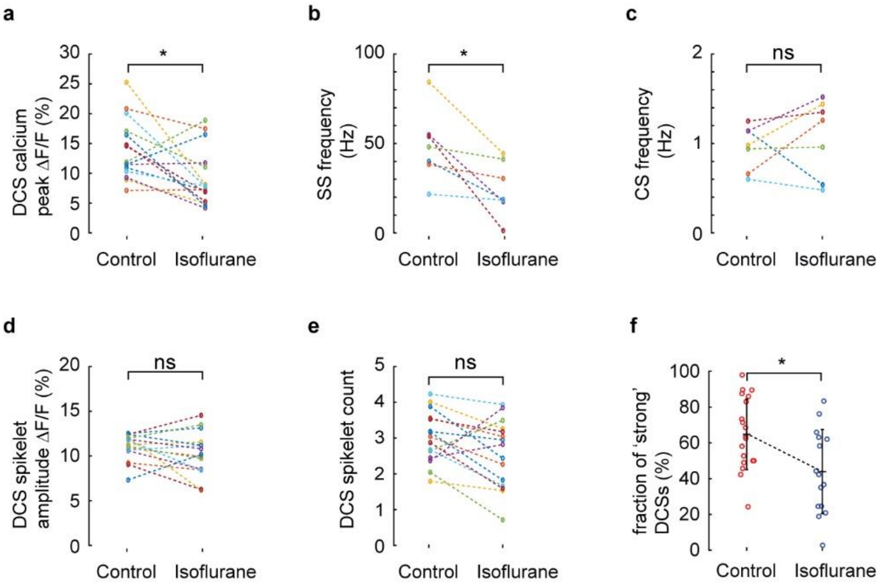

Supplementary Fig. 4 Effect of 1% isoflurane anaesthesia on PN activity. a Average DCS calcium peaks measured in awake (control) and anaesthetized conditions (Isoflurane) from 20 PNs. b Average SS frequency measured in awake and anaesthetized conditions from 7 PNs. c Average CS frequency measured in awake and anaesthetized conditions from 7 PNs. d Average DCS spikelet amplitude measured in awake and anaesthetized conditions from 20 PNs. e Average DCS spikelet counts measured in awake and anaesthetized conditions from 20 PNs. f Average fraction of strong DCSs (with calcium peaks > 5% $\Delta F/F$) measured in awake and anaesthetized conditions from 15 PNs. Bars show mean ± s.d., * $p < 0.05$, ns $p > 0.05$, paired two-tailed t-test.

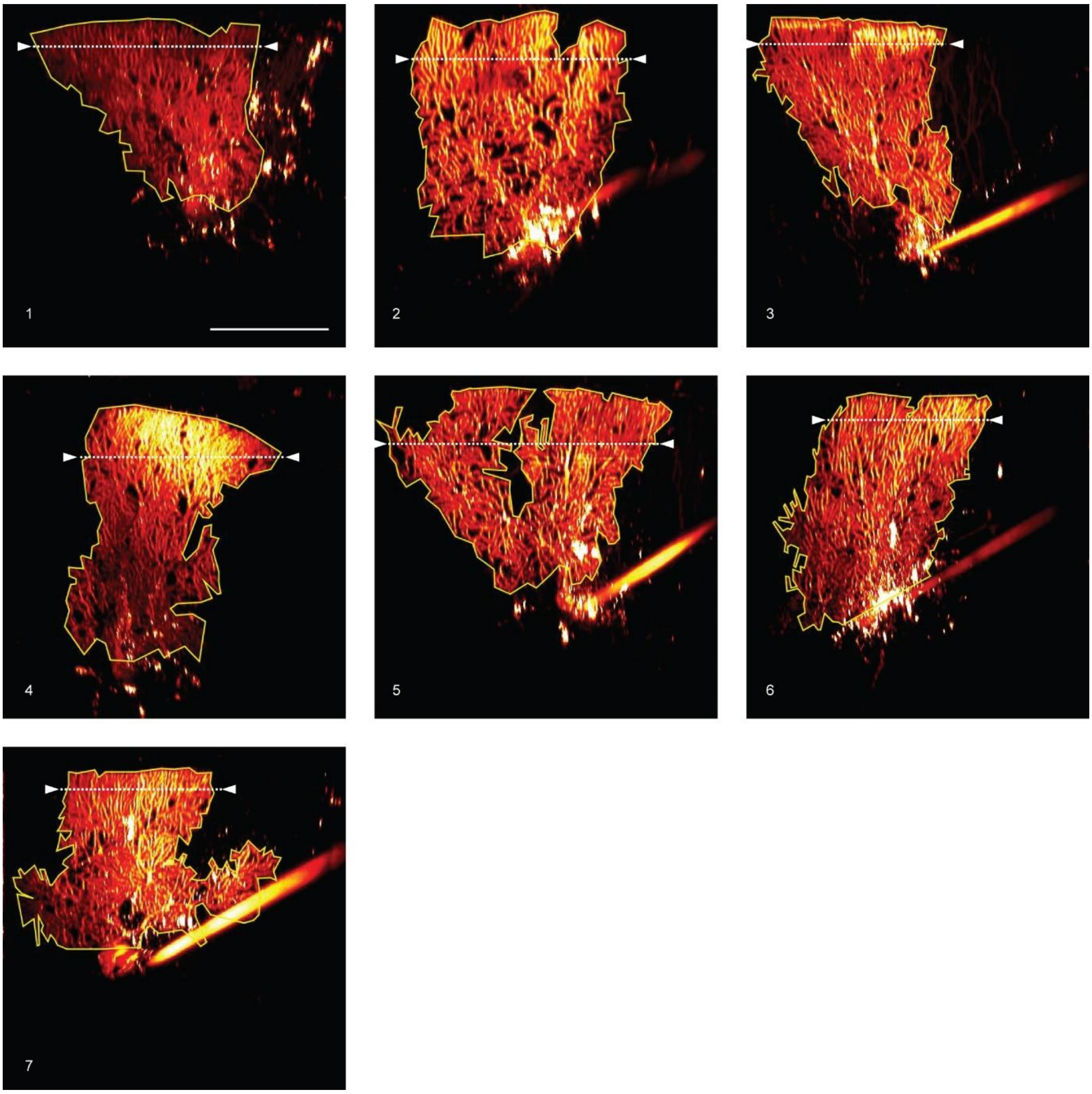

Supplementary Fig. 5 Cerebellar Purkinje neurons labelled in vivo with the voltage-sensitive dye ANNINE6-plus. z-stack projections of 7 PNs showing position of linescan, and the extracellular recording electrode placed at the soma in some examples. Regions selected to calculate approximate dendrite area are outlined (yellow). Scale bar 100μm.

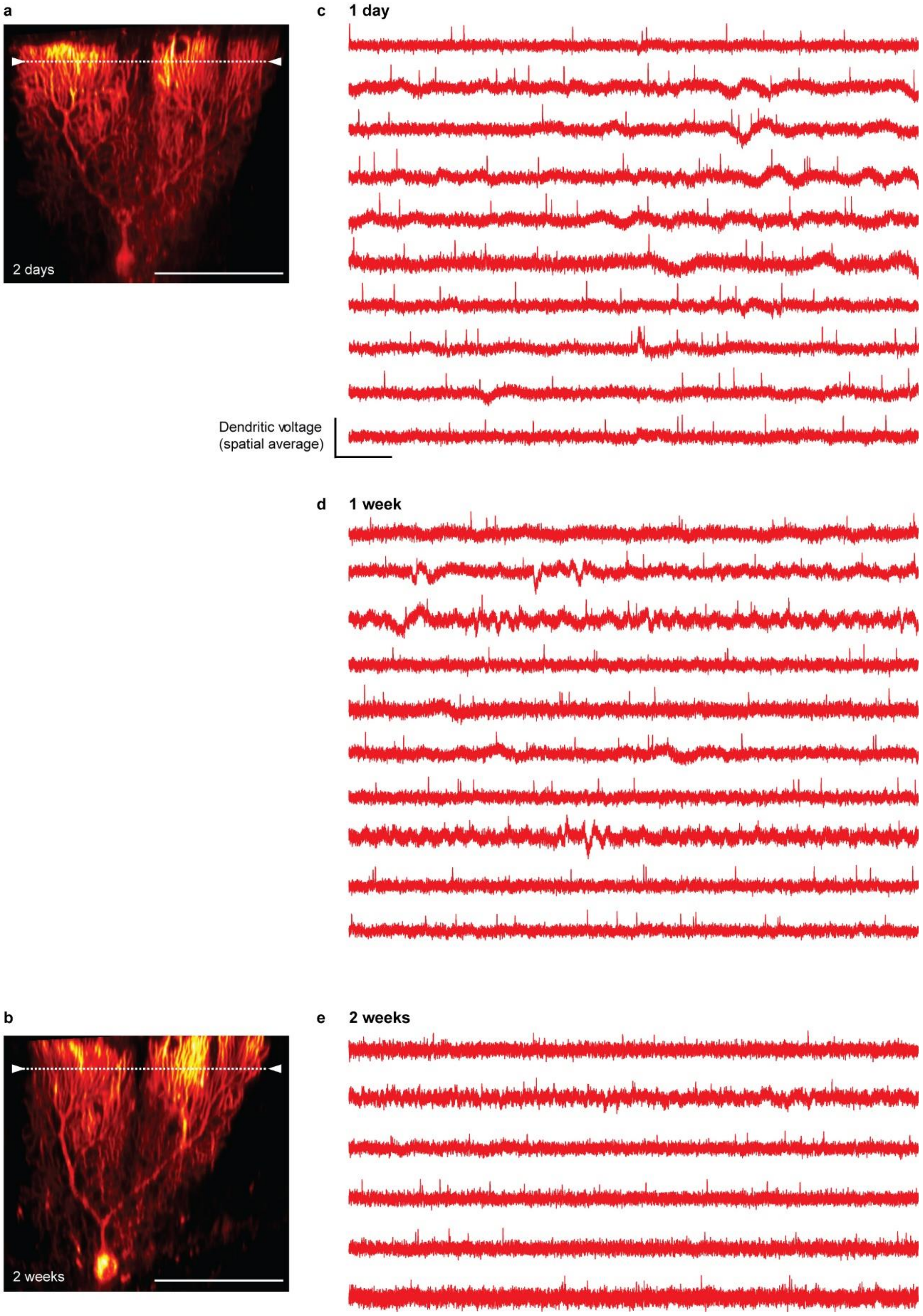

Supplementary Fig. 6 Long-term imaging of dendritic voltage from PNs of awake mice. a, b 3D reconstructions of the same PN made two days and two weeks after labelling with voltage sensitive dye ANNINE-6plus, respectively. Dashed white lines show positions of linescan for dendritic recordings. Scale bars 100μm. Uncorrected dendritic voltage recordings in awake mice made c 1 day, d 1 week, and e 2 weeks after labelling at which point the SNR was reduced. Vertical scale bar 20% ΔF/F; horizontal scale bar 1s.

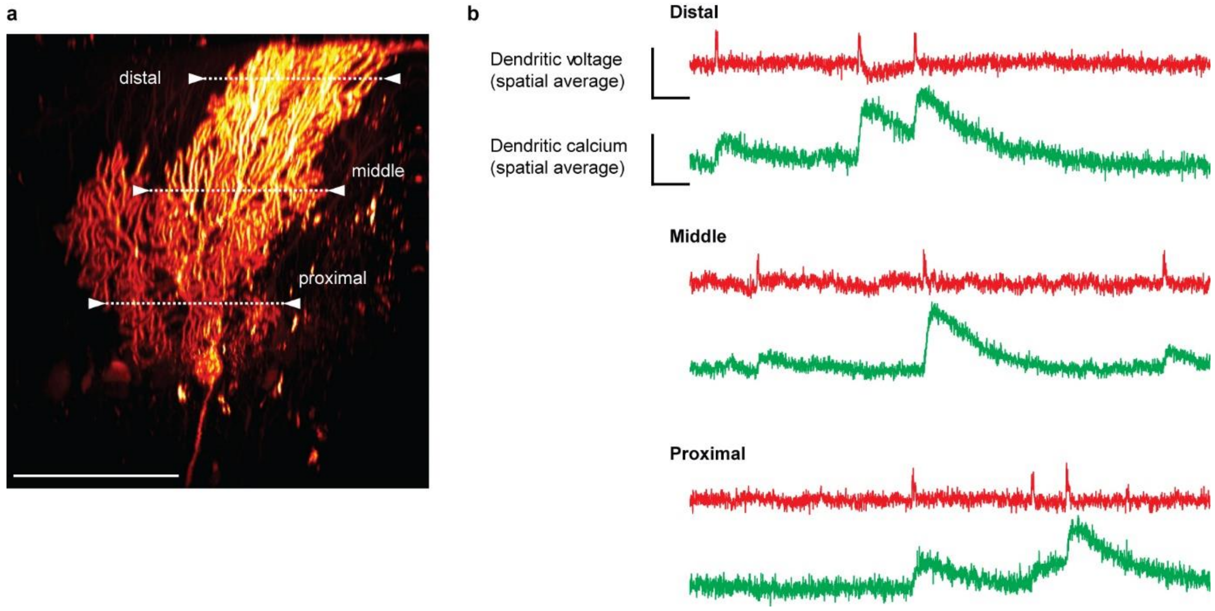

Supplementary Fig. 7 Imaging dendritic voltage at different dendritic locations of the same PNs in an awake mouse. a 3D reconstruction of a PN labelled with voltage sensitive dye ANNINE-6plus and GCaMP6f. Scale bar 100μm. b Dendritic voltage (red traces) and calcium (green traces) recordings made in the distal, middle and proximal dendritic regions of the same PN. All voltage imaging is dominated by structures with high surface to volume ratio, i.e. spiny dendrites. Vertical scale bar 20% ΔF/F; horizontal scale bar 100ms.

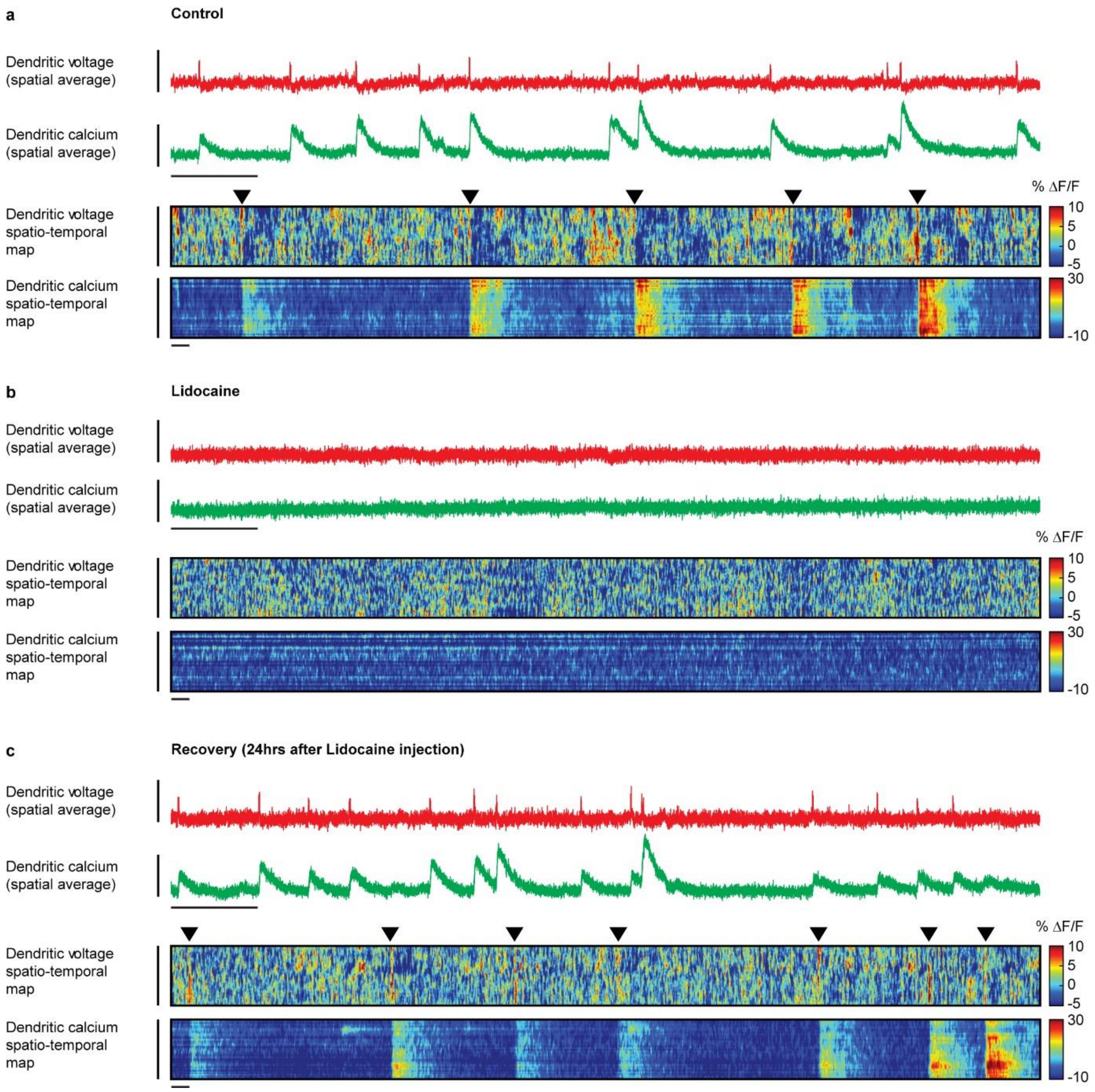

Supplementary Fig. 8 Supra-threshold and sub-threshold dendritic signals are blocked by Lidocaine. a Corresponding traces of spatially averaged dendritic voltage (red traces) and calcium (green traces), (vertical scale bars 20% ΔF/F; horizontal scale bars 1s), and sections of the spatio-temporal map for voltage and calcium (vertical scale bars 50μm; horizontal scale bars 100ms) from the same recording under control conditions, b following lidocaine application, and c 24 hours after lidocaine application, in the same PN all under awake conditions. Filled triangles in the spatio-temporal maps indicate DCS events.

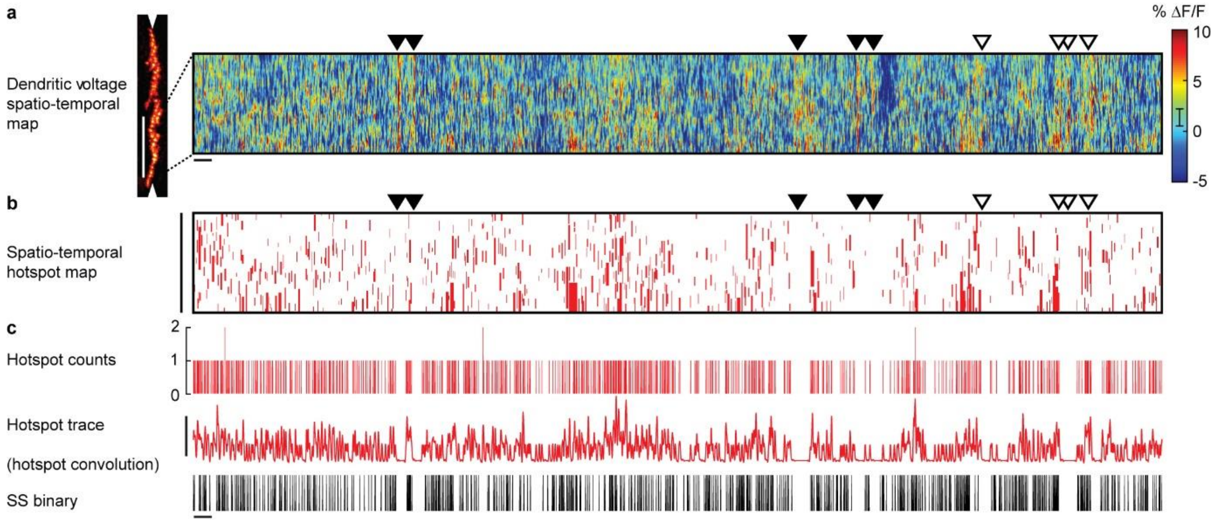

Supplementary Fig. 9 Subthreshold dendritic hotspot activity correlates with somatic SS rate. a Spatio-temporal map for voltage recorded from a 50μm dendrite segment. b Spatio-temporal hotspot map corresponding to a. Vertical scale bars 50μm. c Hotspot counts calculated from b by spatial summation of hotspot events, at 10kHz resolution (interpolated data) (top red bars), corresponding hotspot trace (convolution of hotspot temporal profile and hotspot counts) (middle red trace) and corresponding SS binary trace (bottom black bars), detected using extracellular somatic recording. Vertical scale bar 10% ΔF/F. Filled and open triangles indicate DCS and DS events, respectively, which are excluded from the spatio-temporal hotspot map. Horizontal scale bars 100ms.

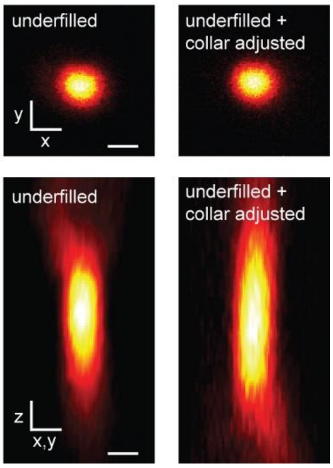

Supplementary Fig. 10 Two-photon excitation volume optimization for voltage imaging in vivo. x-y and z-x, y projections of 1μm fluorescent microspheres used to estimate the 2P excitation volume with 25x/N.A., 1.05 water immersion objective. The bead is similar in size to the diameter of a dendritic shaft. A combination of under filling the objective back aperture and overcompensating for depth with the objective collar, elongates the excitation volume in the z-dimension. Scale bars 1μm.
